# Persistent post-discharge symptoms after COVID-19 in rheumatic and musculoskeletal diseases

**DOI:** 10.1093/rap/rkac008

**Published:** 2022-02-17

**Authors:** Leticia Leon, Ines Perez-Sancristobal, Alfredo Madrid, Leticia Lopez-Pedraza, Jose Ignacio Colomer, Sergio Lerma, Pia Lois, Arkaitz Mucientes, Luis Rodriguez-Rodriguez, Benjamin Fernandez-Gutierrez, Lydia Abasolo

**Affiliations:** 1 Rheumatology Department and IdISSC, Hospital Clinico San Carlos; 2 Health Sciences Faculty, Universidad Camilo Jose Cela; 3 Health Sciences Faculty, Physical Therapy Department, Centro Superior de Estudios universitarios La Salle, Universidad Autonoma de Madrid, Madrid, Spain

**Keywords:** rheumatic diseases, autoimmune diseases, COVID-19, post-acute COVID-19 syndrome, post-acute sequelae, SARS-CoV-2 infection

## Abstract

**Objectives:**

We aimed to describe persistent symptoms and sequelae in patients with rheumatic and musculoskeletal diseases (RMD) after admission owing to coronavirus disease 2019 (COVID-19), assessing the role of autoimmune rheumatic diseases (ARDs) compared with non-autoimmune rheumatic and musculoskeletal diseases (NARDs) on persistent symptoms and sequelae.

**Methods:**

We performed an observational study including RMD patients who attended a rheumatology clinic in Madrid and required admission owing to COVID-19 (between March and May 2020) and survived. The study began at discharge and ran until October 2020. Main outcomes were persistence of symptoms and sequelae related to COVID-19. The independent variable was the RMD group (ARD and NARD). Covariates included sociodemographics, clinical and treatment data. We ran a multivariate logistic regression model to assess the risk of the main outcomes by RMD group.

**Results:**

We included 105 patients, of whom 51.5% had ARD and 68.57% reported at least one persistent symptom. The most frequent symptoms were dyspnoea, fatigue and chest pain. Sequelae were recorded in 31 patients. These included lung damage in 10.4% of patients, lymphopenia in 10%, a central retinal vein occlusion and an optic neuritis. Two patients died. Eleven patients required re-admission owing to COVID-19 problems (16.7% ARD *vs* 3.9% NARD; *P* = 0.053). No statistically significant differences were found between RMD groups in the final models.

**Conclusion:**

Many RMD patients have persistent symptoms, as in other populations. Lung damage is the most frequent sequela. Compared with NARD, ARD does not seem to differ in terms of persistent symptoms or consequences, although ARD might have more re-admissions owing to COVID-19.

Key messages

- After acute coronavirus disease 2019, many patients with rheumatic and musculoskeletal diseases continue to have persistent symptoms, especially fatigue, dyspnoea and chest pain.
- There are no apparent differences between autoimmune rheumatic diseases and non-autoimmune rheumatic and musculoskeletal diseases in terms of persistent symptoms or sequelae.
- Patients with autoimmune rheumatic diseases might have to be re-admitted more frequently because of coronavirus disease 2019.

## Introduction

With the coronavirus disease 2019 (COVID-19) pandemic continuing unabated, the global research community is focusing on short-term treatment and a COVID-19 vaccination strategy. The current pandemic is particularly severe in elderly patients and those with co-morbidities, mainly diabetes, hypertension, ischaemic heart disease, obesity and respiratory diseases [1–5]. In this sense, having a systemic autoimmune condition could be considered an additional risk factor for severity, given that affected patients are more likely to be admitted because of COVID-19, as we recently reported [6].

Previous severe acute respiratory syndrome coronavirus outbreaks have been associated with impaired lung function, muscle weakness, pain, fatigue, depression, anxiety and reduced quality of life after the acute phase [7]. Data on post-acute COVID-19 are scarce [8–12], although COVID-19 is thought to have a major impact on physical, cognitive, mental and social health [13–15]. In a cross-sectional study describing persistence of symptoms of COVID-19 after hospital discharge [12], patients were evaluated an average of 60.3 days after onset of the first symptom of COVID-19. Only 12.6% were completely free of symptoms, while 32% had one or two symptoms and 55% had three or more, particularly fatigue and dyspnoea.

Risk factors and outcomes of the acute phase have been described in patients with rheumatic and musculoskeletal diseases (RMDs) who had overcome COVID-19 [16–18], although little information is available beyond the acute phase. Considering that most patients are chronically ill and complex to manage, it would be interesting to evaluate their clinical course, in addition to the sequelae and the care received after the acute phase of COVID-19.

Our objective was to assess persistent symptoms and sequelae in RMD patients who were discharged from hospital after COVID-19 and to evaluate whether these differed among RMD groups. We also describe the health care received after the acute phase.

## Methods

### Study design and patients

The study was performed in a tertiary hospital of the National Health System of the Community of Madrid, Spain, namely, Hospital Clínico San Carlos (HCSC), which has a catchment area of 350 000 people.

We performed a cross-sectional observational study, including patients from 1 March 2020 (date of the first COVID-19 admission) to 30 May 2020. All patients seen at the rheumatology outpatient clinic of our centre during the study period were preselected.

### Data collection

**Fig. 1:**
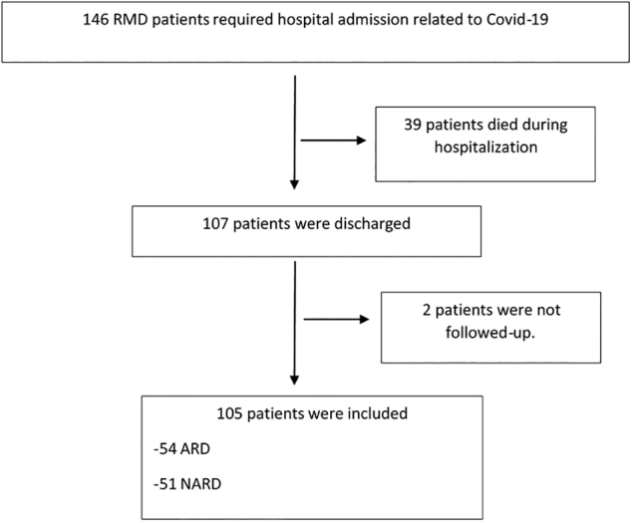
Flow chart for patients until inclusion ARD: autoimmune inflammatory rheumatic diseases; COVID-19: coronavirus disease 2019; NARD: non-autoimmune rheumatic and musculoskeletal disorders; RMD: rheumatic and musculoskeletal disease.

The inclusion criteria were as follows: age >18 years; medical diagnosis of RMD based on the International Classification of Diseases, Tenth Revision (ICD-10); COVID-19 disease confirmed by medical diagnosis or a positive severe acute respiratory syndrome coronavirus 2 (SARS-CoV-2) PCR result and requiring hospital admission during the inclusion period. Follow-up was from discharge (with a maximum end date of 30 May) until 30 October. Patients who died during admission were excluded (Fig. 1).

The study was conducted in accordance with the Declaration of Helsinki and the principles of Good Clinical Practice. In accordance with regulation (EU) 2016/679 of the European Parliament and of the Council of 27 April 2016 on data protection (GDPR) that entered into force on 25 May 2018, the researchers complied with all regulations regarding personal data protection. Patients gave their informed consent for collection of their baseline and pre-COVID-19 clinical data during routine clinical practice and were treated in a department that provides care and carries out research. Regarding the COVID-19-related data, given the nature of the health emergency and the absence of direct intervention in the study patients, the need for informed consent was waived. Following the recommendations of the Spanish Agency of Medicines and Medical Products (Agencia Española de Medicamentos y Productos Sanitarios) regarding the outbreak of SARS-Cov-2 infection, informed consent is not necessary in observational studies (Spanish Ministry of Health, 4 May 2020; reference: MUH 11/2020). The databases were used in accordance with the standards established by the Information Systems Department of the HCSC. This study was approved by the HCSC Institutional Clinical Trials and Ethics Committee (approval number 20/268-E-BS).

### Outcomes

Our primary outcomes were the persistence of symptoms (all new symptoms self-reported by the patient and persisting after discharge) and development of sequelae. These were defined as any new condition resulting from COVID-19 reported in the clinical records or as alterations in diagnostic tests or diagnostic images that persisted after resolution of the acute infection. We included death, lung damage with or without the need for oxygen, analytical abnormalities (lymphocytes, platelets, creatinine and liver markers), thrombotic events and other events related to COVID-19. We also registered re-admission and physical rehabilitation related to COVID-19.

The covariables recorded were as follows: baseline sociodemographic characteristics; RMD group as described in Table 1 [autoimmune rheumatic diseases (ARDs), including systemic autoimmune conditions (SACs) and chronic inflammatory arthritis (CIA); and non-autoimmune rheumatic and musculoskeletal diseases (NARDs)]; long-term therapy before admission [glucocorticoids, NSAIDs, conventional synthetic DMARDs (csDMARDs), targeted synthetic or biologic DMARDs (ts/bDMARDs) including TNF-α agents and other biologics (IL-6 inhibitors, rituximab, abatacept, IL-17/23 inhibitors and Janus kinase inhibitors)]; baseline co-morbid conditions, as described in Table 1; clinical, laboratory and treatment data during admission; and clinical care after discharge (face to face or telephone calls) and the attending physician.

**Table 1:** Baseline sociodemographic and clinical characteristics of rheumatic and musculoskeletal disease patients

Variable	RMD patients admitted with COVID-19 (*n* = 105)
Women, *n* (%)	67 (63.81)
Age, mean (s.d.), years	66.82 (14.83)
Active smoker	3 (2.8)
Diagnosis, *n* (%)	
ARD, *n* (%)	54 (51.4)
SAC	25 (28.8)
** **PMR	6 (5.71)
** **SLE	2 (1.90)
** **MCTD	5 (4.76)
** SjS**	4 (3.81)
** **Vasculitis	1 (0.95)
** **SS	2 (1.90)
** **Polychondritis	1 (0.95)
** **PM	1 (0.95)
** **RP	3 (2.83)
CIA	29 (27.6)
** **RA	19 (18.10)
** **Axial spondyloarthritis	6 (5.71)
** **PsA	3 (2.86)
** **Polyarthritis	1 (0.95)
NARD	51 (48.6)
** **OA	9 (8.57)
** **Tendonitis	17 (16.19)
** **Osteoporosis	2 (1.90)
** **FM	1 (0.95)
** **Low back pain	5 (4.76)
** **Neck pain	1 (0.95)
** **Knee disorder	8 (7.62)
** **Carpal tunnel	1 (0.95)
** **Microcrystalline arthritis	7 (6.67)
Co-morbidities, *n* (%)	
** **Hypertension	32 (30.48)
** **Dyslipidaemia	22 (20.95)
** **Depression	3 (2.86)
** **Diabetes mellitus	10 (9.52)
** **Heart disease^a^	13 (12.38)
** **Ischaemic vascular disease^b^	5 (4.76)
** **Liver disease	7 (6.67)
** **Kidney disease	4 (3.81)
** **Lung disease (ILD/COPD)	15 (14.28)
** **Cancer	6 (5.71)
** **Venous thrombosis/lung embolism	2 (1.90)
** **Thyroid disease	8 (7.62)
Laboratory data at baseline admission, median (IQR)	
** **D-dimer, ng/ml	1147 (451–1395)
** **Lymphocyte count, ×10^9^/l	976 (600–1290)
** **CRP, mg/dl	8.85 (2.62–12.95)
** **Creatinine, mg/dl	0.96 (0.66–1.08)
** **Ferritin, ng/ml	530 (189–718)
COVID-19-related treatments during admission, *n* (%)	
** **HCQ	92 (87.62)
** **Glucocorticoids	59 (56.19)
** **Azithromycin	35 (33.33)
** **Tocilizumab	8 (7.62)
** **Darunavir/cobicistat	5 (4.76)
** **Remdesivir	1 (0.95)
Length of stay, mean (s.d.), days	15.39 (14.42)

a Including: valve disease, arrythmias, cardiomyopathy, heart failure and pericarditis.

b Including: stroke, cardiovascular and peripheral vascular disease.

ARD: autoimmune inflammatory rheumatic diseases; CIA: chronic inflammatory arthritis; COPD: chronic obstructive pulmonary disease; COVID-19: coronavirus disease 2019; ILD: interstitial lung disease; IQR: interquartile range; NARD: non-autoimmune rheumatic and musculoskeletal disorders; RMD: rheumatic and musculoskeletal disease; SAC: systemic autoimmune conditions.

Patient sociodemographic, clinical, laboratory and treatment data were obtained through the clinical records of the Rheumatology Department of HCSC. Admission and post-discharge data were collected from the information systems of the HCSC and primary health care. All patient information, including co-morbidities, clinical data, laboratory findings, imaging findings (chest X-rays or scans), treatment and main outcomes, were checked and evaluated by two rheumatologists from the research team.

### Statistical analysis

Patient characteristics are expressed as the mean and SD or median and interquartile range for continuous variables; categorical variables are expressed as percentages. For the comparison of persistent symptoms and sequelae between ARD and NARD, we used the Mann–Whitney *U*-test, χ^2^ test or Fisher exact test, as appropriate. Multivariate logistic regression models (adjusted for age, sex and co-morbidity) were run to examine the possible effect of RMD group on outcome (persistent symptoms or sequelae attributable to COVID-19). The model also included variables with *P* < 0.2 in the univariate regression analysis. The results were expressed as the odds ratio (OR) with its respective 95% CI. All analyses were performed using STATA v.13 (Stata Corp., College Station, TX, USA). A two-tailed *P*-value < 0.05 was considered to indicate statistical significance.

## Results

A total of 146 RMD patients required admission to hospital during the study period; of these, 107 survived and were discharged. Two were excluded (no information in data sources). The final study population comprised 105 patients (Fig. 1), and follow-up ranged from 4 to 7 months.

Sociodemographic and clinical characteristic are shown in Table 1. Two-thirds of the patients were women, and the mean age was 66.82 (14.83) years. The mean duration of RMD was 8.9 (8.2) years, and the mean duration of admission owing to COVID-19 was 15.38 (14.42) days. Slightly more than half (51.43%) had ARD, with RA being the main diagnosis. In patients with NARD, the most frequent findings were tendonitis and OA. Interestingly, 46.15% of the patients had at least one co-morbid condition. Regarding long-term therapy before admission, 32% of the patients were taking glucocorticoids and 19% were taking NSAIDs. The treatments taken by patients with ARD were as follows: antimalarials, 8 (14.8%); MTX, 27 (50%); AZA, 5 (9.25%); LEF, 4 (7.4%); SSZ, 3 (5.5%); and bDMARDs (rituximab = 3; tocilizumab = 1 and TNF-α = 3), 7 (12.9%).

During admission, 92.5% of patients had pneumonia, 41% had complications related to COVID-19 (sepsis, thrombosis, ischaemic, cardiac and renal), and 40% had lymphopenia (lymphocyte count <0.8 × 10^9^/l). Therapy was mainly with antimicrobials and CSs.

After discharge, all the patients were followed up by at least one physician; 80 (85%) were followed by their primary care physician (telephone calls) and 45 (42%) by a specialist (face to face), mainly a pneumologist (*n* = 22) or internist (*n* = 9). In addition, 17 ARD patients were managed by rheumatologists through telephone calls. Analytical blood tests were performed in 89 patients and X-rays in 77.

Regarding persistent symptoms after discharge (Table 2), 68.57% of patients had at least one symptom, and 26.84% had more than three. In those with symptoms, the most frequently reported were dyspnoea, fatigue, chest pain, cough and diarrhoea/vomiting. All lasted on average >1 month, with diarrhoea and fever having the shortest duration. The longest reported duration was >3 months for alopecia and physical deconditioning.

**Table 2:** Symptoms and sequelae in RMD patients discharged after COVID-19

Symptoms and sequelae	*n* (%)	Duration, median (IQR), days
Self-reported symptoms (*n* = 105)	72 (68.57)	
Dyspnoea	38 (36.2)	68.5 (23–97.5)
Fatigue	27 (25.71)	38.5 (14–83)
Chest pain	18 (17.14)	30 (14–150)
Cough	14 (13.33)	27 (18–99)
Diarrhoea/vomiting	14 (13.33)	31 (15–55)
Fever	11 (10.48)	29 (22–48)
Anosmia/dysgeusia	9 (8.57)	92 (25–134)
Anxiety/depression	8 (7.62)	56.5 (22.5–73)
Dermatological manifestations	7 (6.67)	37 (27–68)
Joint pain	6 (5.71)	55 (20–68)
Visual/auditory alterations	5 (4.76)	53 (18–112)
Headache	5 (4.76)	28 (12.5–85.5)
Physical deconditioning	4 (3.81)	111 (45.5–138.5)
Hair loss	2 (1.90)	83.5 (83–84)
Cognitive dysfunction	1 (0.95)	20
Sequelae (*n* = 105)	31 (29.5)	
Death	2 (1.90)	13.2 (8–19)
Analytical abnormalities (*n* = 89)	16 (17.97)	45.5 (21–81)
Lung damage	12 (10.47)	44 (24–77.5)
Need for oxygen	6 (5.7)	20.5 (7–47)
Thrombotic event	1 (0.95)	42
Optic neuritis	1 (0.95)	27
Rehabilitation needed (physical/respiratory)	5 (4.76)	54 (43–88)

The median duration of symptoms was the lag time from discharge until the last date the event was reported. The median duration of sequelae was the lag time from discharge until the sequela was reported.

IQR: interquartile range.

**Fig. 2:**
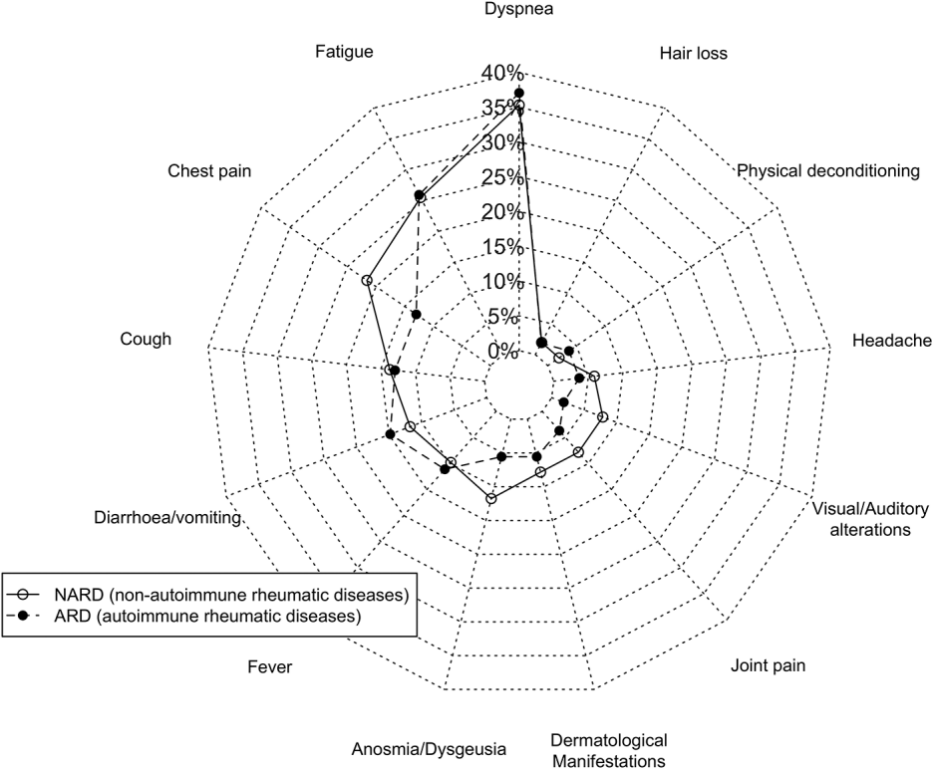
Persistent symptoms after COVID-19 ARD: autoimmune inflammatory rheumatic diseases; NARD: non-autoimmune rheumatic and musculoskeletal disorders.

We did not find statistically significant differences in persistent symptoms between ARD (68.5%) and NARD (68.6%) patients. The mean number of symptoms was 1.48 (1.46) for ARD and 1.64 (1.65) for NARD, although the difference was not significant. As we show in Fig. 2, the types of persistent symptoms were very similar among ARD and NARD patients, with slight differences recorded for chest pain (*P* = 0.3), gastrointestinal symptoms (*P* = 0.3), anosmia/dysgeusia (*P* = 0.3) and visual/auditory abnormalities (*P* = 0.2).

During the post-discharge follow-up, 29 patients returned 31 times to the emergency department and 14 (50%) required admission, which was related to COVID-19 in 78.6% of cases (*n* = 11) [mean and median lag time, 31.6 (28.5) and 19 (8–53) days]. The reasons were mainly lung problems, thrombotic problems, kidney failure, hepatitis and cutaneous anaphylaxis. One ARD patient was re-admitted with COVID-19 reinfection. We found other cases of COVID-19 reinfection after 5 months that did not require admission (NARD). Interestingly, COVID-19-related re-admissions were more common in ARD patients (16.7%; 33% owing to lung or kidney infections) than in NARD patients (3.9% and no infections), with the difference almost reaching statistical significance (*P* = 0.053).

Regarding the 77 patients who underwent radiological imaging during follow-up, 75 improved gradually over time [mean and median lag time, 61 (29) and 54 (41–74) days], and 20 (26.6%) achieved complete radiological recovery during the follow-up period. In two cases, the condition of the patient worsened (one developed a lung cancer and the other pneumothorax). Six patients required oxygen support because of lung damage after COVID-19, with similar results in both RMD groups.

**Fig. 3:**
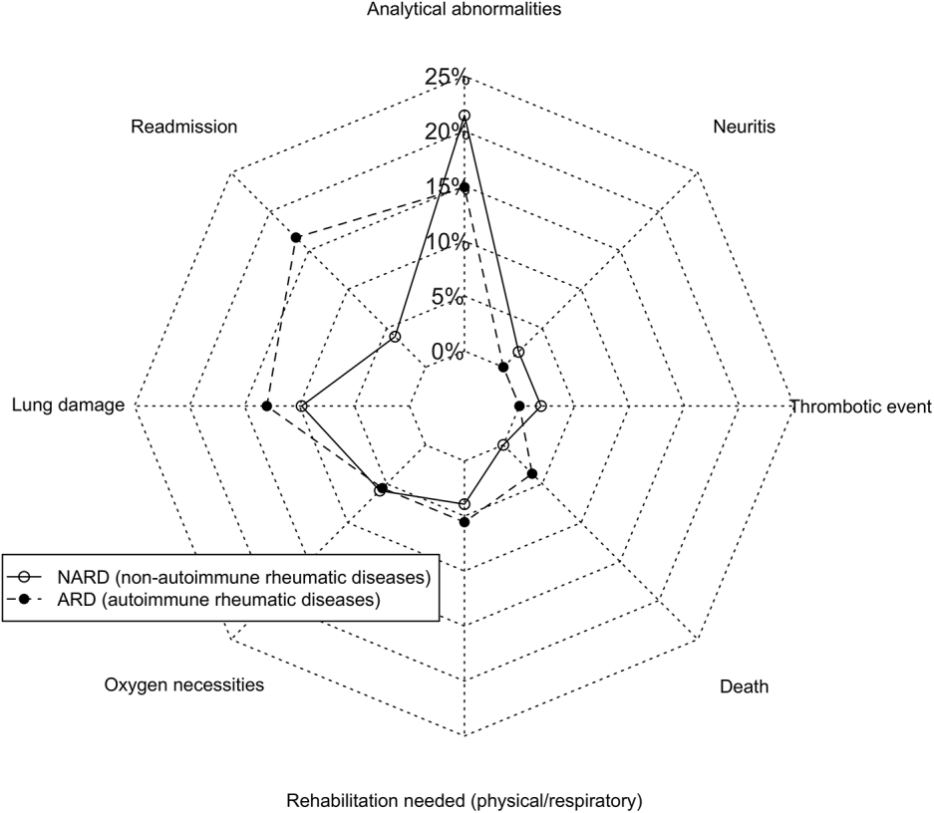
Sequelae after COVID-19 ARD: autoimmune inflammatory rheumatic diseases; NARD: non-autoimmune rheumatic and musculoskeletal disorders.

We considered lung damage to be a sequela based on the radiographic image, pulmonary function tests, clinical course and oxygen support after discharge. Twelve patients (11.4%) were considered to have lung damage over time (Table 2; Fig. 3): two with bacterial infection requiring re-admission [mean time: 29 (20.5) days], one with lung cancer (after 74 days), three with previous chronic obstructive pulmonary disease that impaired baseline function [one developed atelectasis; mean time, 43.5 (10.5) days] and six with residual interstitial pneumonitis [mean time, 89.2 (53) days]. One of the six patients with pneumothorax required hospital admission at least three times during the study period (the first 8 days after discharge) and another had previous interstitial lung disease that worsened after the acute infection. Although ARD patients more commonly experienced lung damage, the differences were not statistically significant (*P* = 0.7).

Blood tests revealed that 18% of patients developed abnormalities that were not present before admission: nine (10%) with lymphopenia (lymphocyte count <0.8 × 10^9^/l), four with elevated liver transaminases, five with renal failure and two with thrombopenia. Blood test abnormalities were observed in 14% of patients with ARD compared with 21% of those with NARD, although the difference was not significant (*P* = 0.4). In the case of lymphopenia, blood test abnormalities were detected in 11% of cases of ARD and 5.9% of cases of NARD (*P* = 0.4).

Two patients died during follow-up. Both had ARD, and their median age was 87.5 (84–91) years. One had relevant co-morbidity, including previous lung disease, and died at home 8 days after discharge. The other patient died of urosepsis during re-admission 87 days after discharge.

Regarding other sequelae, one patient developed a central retinal vein occlusion and another optic neuritis. Both were NARD patients (Fig. 3). Finally, intensive rehabilitation was necessary in five patients, four of whom had associated physical deconditioning symptoms. Overall, 31 patients had at least one sequela resulting from COVID-19. The prevalence was similar between the groups (ARD: 29.6%; NARD: 29.4%; *P* = 0.9).

The risk of persistent symptoms was lower in ARD than in NARD patients, although the difference was not statistically significant in the univariate analysis [OR: 0.99 (0.43–2.27)] or in the multivariate analysis (Table 3). In the latter, prior co-morbidities, female sex and younger age were associated with a greater probability of post-discharge symptoms. The final model also revealed statistically significant differences for other factors, such as lymphopenia and pneumonia during admission.

**Table 3:** Multivariate analysis. Risk factors for persistent symptoms after COVID-19 in RMD. Independent variable RMD group

Variable	OR	95% CI	*P*-value
Sex, female	3.53	1.24, 10.08	0.01
Age, years			
<50–60	1	–	−
60–75	0.11	0.02, 0.48	0.004
>75	0.09	0.02, 04	0.002
ARD *vs* NARD	0.43	0.15, 1.23	0.12
Baseline number of co-morbidities^a^	1.90	1.09, 3.31	0.023
Lymphopenia^b^	3.17	1.07, 9.4	0.038
Pneumonia^c^	10.1	1.5, 66.01	0.017

a Interstitial lung disease/chronic obstructive pulmonary disease.

b Lymphopenia: lymphocyte count <0.8 × 10^9^/l during admission.

c Presence of pneumonia during admission. Defined as the number of co-morbidities before admission with coronavirus disease 2019, including the following: hypertension, heart disease, ischaemic vascular disease, diabetes mellitus, venous thrombosis/lung embolism, chronic kidney disease, liver disease and lung disease (interstitial lung disease/chronic obstructive pulmonary disease).

ARD: autoimmune inflammatory rheumatic diseases; NARD: non-autoimmune rheumatic and musculoskeletal disorders; OR: odds ratio; RMD: rheumatic and musculoskeletal disease.

Concerning the influence of RMD on sequelae, no differences were found between ARD and NARD in the univariate analysis or in the multivariate analysis [OR: 1.85 (0.67–5.14); *P* = 0.23]. Likewise, there were no differences between age groups or for baseline co-morbidity, although women were less likely to experience sequelae [OR: 0.28 (0.10–0.77); *P* = 0.014]; this risk increased if they had experienced complications of COVID-19 during admission [OR: 9.54 (3.16–28.8); *P* = 0.001].

## Discussion

We found that symptoms persisted in a higher number of RMD patients after admission owing to COVID-19, even 6 months after discharge. The most common symptoms were fatigue, dyspnoea, anosmia and chest pain. Our study also evaluated sequelae, highlighting that lung damage seems to be the most frequent in these patients. There were no apparent differences in terms of persistent symptoms or sequelae, although the number of re-admissions seems to be higher in ARD patients.

Available data regarding persistent symptoms after COVID-19 are scarce and heterogeneous, although they all indicate that this is a frequent issue [8, 11]. In our study, almost 70% of patients reported persistence of at least one symptom.

Almost 40% of the study patients reported dyspnoea, with an average duration of >2 months. This was the most frequent persistent symptom, as reported elsewhere [11, 13, 19]. Our results for persistent fatigue, cough and gastrointestinal symptoms are also in line with published data [11, 13, 20].

The frequency of persistent joint and muscle pain has been reported to be ∼10–27% at 4–8 weeks after infection [11, 13, 19]. Our follow-up is similar, although the percentages found are lower, probably because most of the RDM patients had previously experienced this symptom and did not report it as a sequela of COVID-19.

In the case of other symptoms, such as anosmia, headache, physical deconditioning, anxiety or depression, we found lower percentages than in other studies [11, 13, 19, 21–24]. Nevertheless, we must take into account that our data were collected retrospectively from medical records and patients were not asked specifically for them. Those symptoms might not have been reflected in the clinical history because they were considered less relevant than others for the physician or the patient or were symptoms that the patient did not report because they had been insufficiently bothersome.

Residual interstitial abnormalities of COVID-19 pneumonia persisted in some patients for 3 months. In others, chronic obstructive pulmonary disease had worsened over 2 months, suggesting that lung damage might persist in some discharged patients. Other studies have also reported a significant percentage of patients with abnormal results in respiratory function and imaging studies after acute COVID-19 infection [20, 25, 26]. Almost 12% of the RMD patients in this study developed lung disease or their lung damage worsened after discharge, accounting for 39% of the total sequelae we recorded.

Consistent with our findings for thrombotic events, other studies reported a prevalence of 0.5–2.5% [27, 28]. We reported one ocular thromboembolic event, as reported elsewhere [29].

During the follow-up, 27.6% of the patients returned to the emergency department owing to a health problem. Of these, 50% had to be re-admitted during the first 30 days after having overcome COVID-19. In the study by Donnelly *et al.* [30], the percentage of re-admissions was somewhat higher. The study by Donnelly *et al.* [30] was carried out within the general population; however, many of our RMD patients continued receiving their regular appointments (face to face or virtual). These circumstances might mean that some of the post-discharge problems were detected and treated earlier, avoiding re-admissions [30].

Four discharged patients received chest physiotherapy. Although this number seems reasonable considering the patients who manifested deconditioning, it is low when compared with the presence of dyspnoea. In this sense, more attention should be given to the management of the post-COVID-19 patient. Patients receiving oxygen support would benefit from low-intensity exercise, according to the recommendations of the Stanford Hall consensus statement for post-COVID-19 rehabilitation [31].

Little is known about musculoskeletal impairments related to post-COVID-19. In patients who have been deconditioned for long periods, muscles and joints can worsen without physical therapy; this is particularly true for patients with previous RMD. Aerobic exercise and adapted physical activity are considered the main beneficial interventions [32]. In the case of hospitalized patients with co-morbid conditions, long stays and the need for intensive care, rehabilitation would be mandatory.

Despite the excess patient mortality during hospital admission at the peak of the pandemic, 1.9% of the patients died of COVID-19 in the first month after hospital discharge, although no further deaths were recorded.

Concerning the influence of RMD on persistent symptoms, the risk for ARD was lower, albeit without statistical significance. This slight difference could be related to the tight control of patients with ARD, including strict management of co-morbidities, especially cardiovascular risk factors with statins [33, 34] or osteoporosis with vitamin D [35]. However, it might also be because of therapy with DMARDs, which might have a beneficial effect with respect to complications of COVID-19. Another interesting finding is that female sex, younger age and the presence of lymphopenia or pneumonia during admission were independently associated with persistent symptoms. In accordance with the results of Cook *et al.* [36], our study showed that baseline co-morbidities can increase the risk of persistent symptoms.

Analysis of sequelae did not reveal differences between ARD and NARD patients, although male sex and COVID-19-related complications during admission increased the risk. However, the number of re-admissions was higher in ARD patients, probably because of the risk of infections and complications associated with immunosuppression.

Post-infection care for COVID-19 survivors increases the burden on the health system, which was considerably strained by the challenges of the pandemic. Affected patients were closely followed up after hospitalization, especially ARD patients, and the role played by rheumatology nurses was particularly important.

### Limitations

Our study has several limitations. Given that it is a retrospective observational study, we cannot be sure that all relevant data were reported. Furthermore, it is cross-sectional and, as such, unable to reflect how the problems evolve over time. In this sense, longitudinal studies designed specifically to assess the course of post-COVID-19 patients over time would be necessary to gain a better understanding of the progression of symptoms and sequelae.

Notwithstanding these limitations, we believe that it is necessary to report real-world data after the acute phase of COVID-19 in RMD. In this sense, our study provides an overview of the situation of RMD patients covering a period of ≤7 months. Although these findings are preliminary, we provide both rheumatologists and ARD patients with some reassurance, because the disease course seems no worse in this group than in that of other post-COVID-19 patients, except for the higher percentage of re-admissions found in ARD. Consequently, closer monitoring would be advisable. Our study points to factors that could play a role in the post-COVID-19 phase.

### Conclusions

To conclude, we report on symptoms and sequelae after hospitalization because of COVID-19 in RMD patients. This study can be considered a starting point from which we can extend our knowledge. Longer follow-up studies in larger RMD cohorts are necessary to understand the full spectrum of the health consequences of COVID-19 and to design future care plans.

Our findings indicate that many RMD patients continue to have symptoms (especially fatigue, dyspnoea and chest pain) after acute COVID-19 infection. There seem to be no differences between ARD and NARD in terms of persistent symptoms or sequelae, although ARD patients might have to be re-admitted more frequently because of COVID-19.
